# Supplementary material for: The impact of pyrethroid resistance on the efficacy and effectiveness of bednets for malaria control in Africa
Source: eLife. 2016 Aug 22;5:e16090. doi: 10.7554/eLife.16090 (PMC5025277; doi:10.7554/eLife.16090)
Supplement: Figure 2—source data 1. — Different search engines were used on different dates (each with their own search numbering) DOI: http://dx.doi.org/10.7554/eLife.16090.008 [file elife-16090-fig2-data1.docx]

**Supplementary Tables**

**Table 2–table supplement 1. Summary of the different predefined search strings used for meta-analysis *M1* - *Predicting LLIN effectiveness from bioassay mortality*** - PubMed search 20/05/2015.

| Search | Query | Items found |
| --- | --- | --- |
| 3 | Search (#1 AND #2) | 230 |
| 2 | Search hut*[Title/Abstract] | 5778 |
| 1 | Search (Mosquito[Title/Abstract]) OR anopheles[Title/Abstract] | 26855 |

**Table 2–table supplement 2. Summary of the different predefined search strings used for meta-analysis *M2 - Estimating the impact of PBO in pyrethroid bioassays****.* Different search engines were used on different dates (each with their own search numbering)

| Search | Query | Items found |
| --- | --- | --- |
| PubMed search string 25/02/2015 | |  |
| 13 | Search (#3 AND #7 AND #12) | 79 |
| 12 | Search (#8 OR #9 OR #10 OR #11) | 955591 |
| 11 | Search pyrethroid resist*[Title/Abstract] | 661 |
| 10 | Search insecticide resist*[Title/Abstract] | 1959 |
| 9 | Search susceptib*[Title/Abstract] | 304538 |
| 8 | Search resist*[Title/Abstract] | 730778 |
| 7 | Search (#4 OR #5 OR #6) | 2261 |
| 6 | Search synergist[Title/Abstract] | 674 |
| 5 | Search piperonyl butoxide[Title/Abstract] | 965 |
| 4 | Search PBO[Title/Abstract] | 1005 |
| 3 | Search (#1 OR #2) | 26431 |
| 2 | Search anopheles[Title/Abstract] | 10418 |
| 1 | Search mosquito[Title/Abstract] | 20563 |
|  |  |  |
| Ovid Medline (R) search string 04/03/2015. | |  |
| 1 | mosquito.ab. or mosquito.ti. or anopheles.ab. or anopheles.ti. | 24412 |
| 2 | PBO.ab. or PBO.ti. or piperonyl butoxide.ab. or piperonyl butoxide.ti. or synergist.ab. or synergist.ti. | 1902 |
| 3 | resist*.ab. or resist*.ti. or susceptib*.ab. or susceptib*.ti. or insecticide resist*.ab. or insecticide resist*.ti. or pyrethroid resist*.ab. or pyrethroid resist*.ti. | 865839 |
| 4 | 1 and 2 and 3 | 68 |
|  |  |  |
| Scopus search string 04/03/2015. | |  |
| 1 | (TITLE-ABS-KEY(mosquito) OR TITLE-ABS-KEY(anopheles)) AND (TITLE-ABS-KEY(PBO) OR TITLE-ABS-KEY(piperonyl butoxide) OR TITLE-ABS-KEY(synergist)) AND (TITLE-ABS-KEY(resist*) OR TITLE-ABS-KEY(susceptib*) OR TITLE-ABS-KEY(insecticide resist*) OR TITLE-ABS-KEY(pyrethroid resist*)) | 135 |
|  |  |  |
| Web of Science search string 05/03/2015 | |  |
| 1 | TI=(mosquito OR anopheles) AND TI=(PBO OR piperonyl butoxide OR synergist) AND TI=(resist* OR susceptib* OR insecticide resist* OR pyrethroid resist*) | 8 |
|  |  |  |
| PubMed search string 06/03/2015 | |  |
| 4 | Search (#1 AND #2 AND #3) | 143 |
| 3 | Search (mosquito[Text Word]) OR anopheles[Text Word] | 31327 |
| 2 | Search ((PBO[Title/Abstract]) OR synergist[Title/Abstract]) OR piperonyl butoxide[Title/Abstract] | 2263 |
| 1 | Search ((PBO[Text Word]) OR piperonyl butoxide[Text Word]) OR synergist[Text Word] | 2432 |

**Table 2–table supplement 3. Summary of the different predefined search strings used for meta-analysis *M3 - Estimating the impact of PBO in experimental hut trials.*** PubMed search string (12/03/15).

| Search | Query | Items found |
| --- | --- | --- |
| 5 | Search (#1 AND #2 AND #3 AND #4) | 20 |
| 4 | Search (resist* OR susceptible* OR insecticide resist* OR pyrethroid resist*) Title/Abstract | 816838 |
| 3 | Search (PBO OR piperonyl butoxide OR synergist) Title/Abstract | 2265 |
| 2 | Search (mosquito OR *anopheles*) Title/Abstract | 26479 |
| 1 | Search (nets OR bednets OR ITN OR LLIN OR mosquito net OR PermaNet OR Olyset) Title/Abstract | 7010 |
