# Supplementary material for: The impact of pyrethroid resistance on the efficacy and effectiveness of bednets for malaria control in Africa
Source: eLife. 2016 Aug 22;5:e16090. doi: 10.7554/eLife.16090 (PMC5025277; doi:10.7554/eLife.16090)
Supplement: Source code 1. — DOI: http://dx.doi.org/10.7554/eLife.16090.028 [file elife-16090-code1.docx]

**Source Code**

The following OPENBUGS code was used to fit the best fit functional relationships shown in Figure 2.

model{

##the relationship between bioassay mortality with and without PBO for different mosquito species (Figure 2B)

for(mrs in 1:n_assays){ my_assay_rand[mrs]~dnorm(0,tauA)} ##Random effects

for(v in 1:n_points) {c_dead[v]~dbin(hp[v],c_n[v])

pbo_dead[v]~dbin(gp[v],pbo_n[v])

my_funG[v]<-k[1]+(k[2]*(hp[v]-0.5))/(1+(hp[v]-0.5)*k[3])

my_funF[v]<-k[4]+(k[5]*(hp[v]-0.5))

my_fun[v]<-step(-abs(species[v]-2))*my_funF[v]+step(-abs(species[v]-1))*my_funG[v]

logit(mf[v])<-my_fun[v]+my_assay_rand[assays[v]]

gp[v]<-max(0,min(mf[v],1))

hp[v]~dunif(0,1)}

##the relationship between assay mortality and hut trial mortality (Figure 2A)

for(mr in 1:n_study){my_rand[mr]~dnorm(0,tau)} ##Random effects

for (a in 1:n_convert) {assay_dead[a]~dbin(ap[a],assay_N[a])

hut_dead[a]~dbin(bp[a],hut_N[a])

af[a]<-k[6]+k[7]*(ap[a]-0.5)+my_rand[assay_study[a]]

logit(bp[a])<-af[a]

ap[a]~dunif(0,1)}

tauA<-k[8] tau<-k[9]

##Priors

k[1] ~dnorm(0,0) k[2] ~dnorm(0,0) k[3]~dnorm(0,0) k[4]~dnorm(0,0) k[5]~dnorm(0,0) k[6]~dnorm(0,0) k[7]~dnorm(0,0) k[8]~dnorm(0,0) k[9]~dnorm(0,0)}

The following OPENBUGS code was used to fit the best fit functional relationships shown in Figure 3.

model{

for (s in 1:n_sites) {mean_caught_untreated[s]~dnorm(my_caught,tau_site)

site_ind_s[s]~dnorm(0,tau_site_s) } ##Random effects

## Relationship between hut trial mortality and deterrence (Figure 3A)

for (v in 1:n_points) {mean_caught[v]<-is_treat[v]*mean_caught_untreated[my_site_u[v]]*(1-my_det[v])+(1-is_treat[v])*mean_caught_untreated[my_site_u[v]]

my_det[v]<-k[4]+k[5]*(hp[v]-0.5)+k[6]*pow((hp[v]-0.5),2)

caught_u[v]~dnorm(mean_caught[v],tau_caught)

prop_hp[v]<-dead_u[v]/caught_u[v]

dead_u[v]~dbin(hp[v],caught_u[v])

hp[v]~dunif(0,1)}

## Relationship between hut trial mortality and feeding success (Figure 3C)

for (x in 1:26) {my_function[x]<-k[7]*(exp(k[8]*(1-hp[x]))-1)+site_ind_s[my_site_u[x]]

my_all[x]<-min(1,max(0,my_function[x]))

suc_u[x]~dbin(my_all[x],caught_u[x])

prop_u[x]<-suc_u[x]/caught_u[x]}

## Relationship between number of washes and LLIN halflife

for (w in 1:n_points2) {dead_w[w]~dbin(decay_mort_all[w],caught_w[w])

decay_mort_tog[w]<-hp[n_omit[w]]

decay_mort_all[w]<-min(1,max(0,decay_mort_tog[w]*decay_mort[w]))

logit(decayM[w])<-k[9]+(hp[n_omit[w]])*k[10]

decay_mort[w]<-exp(-washes[w]*decayM[w])}

tau_site<-pow(k[1],-1) tau_caught<-pow(k[2],-1) my_caught<-k[3] tau_site_s<-pow(k[11],-1)

##Priors

k[1] ~dgamma(0.0000001,0.0000001) k[2] ~dgamma(0.0000001,0.0000001) k[3]~dgamma(0.0000001,0.0000001) k[4]~dnorm(0,0) k[5]~dnorm(0,0) k[6]~dnorm(0,0) k[7]~dgamma(0.0000001,0.0000001) k[8]~dgamma(0.0000001,0.0000001) k[9]~dnorm(0,0) k[10]~dnorm(0,0) k[11]~dgamma(0.0000001,0.0000001)}
